# Thermodynamic, Exergy, and DFT-Based QTAIM Analysis of R452A Refrigerant: A Multiscale Molecular–System Approach

**DOI:** 10.3390/molecules31122071

**Published:** 2026-06-12

**Authors:** Hacer Gümüş, Sezgin Büyükkütük, Arzu Keven

**Affiliations:** 1 Automotive Technology Program, Golcuk Vocational School, Kocaeli University, 41380 Kocaeli, Turkey; arzu.keven@kocaeli.edu.tr; 2 Mechanics, Golcuk Vocational School, Kocaeli University, 41650 Kocaeli, Turkey; sezgin.buyukkutuk@kocaeli.edu.tr

**Keywords:** R452A, refrigerant, thermodynamic analysis, topological analysis, DFT

## Abstract

In this study, the R452A refrigerant used in refrigerated trucks is investigated through a multiscale approach combining thermodynamic and molecular-level analyses. The performance of the vapor compression refrigeration system is evaluated using energy and exergy analyses to assess system efficiency and identify irreversibilities. At the molecular level, Density Functional Theory (DFT) is employed to investigate the electronic structure and bonding characteristics of refrigerant components. This approach enables a detailed understanding of molecular properties that influence macroscopic thermodynamic behavior in refrigeration systems.

## 1. Introduction

Refrigeration systems play a crucial role in food and pharmaceutical cold-chain transportation, where temperature stability directly determines product quality [1], safety, and shelf life [2]. Vapor compression refrigeration systems used in refrigerated vehicles exhibit varying performance depending on operating conditions [3] and the efficiency of cycle components. In this context, exergy analysis provides a powerful tool for evaluating energy degradation and identifying thermodynamic irreversibilities within the system [4].

The vapor compression refrigeration cycle is one of the most widely used cooling technologies, applied in domestic refrigeration, air conditioning, and refrigerated transport. Its primary function is to transfer heat from a low-temperature region to a higher-temperature environment through mechanical work input.

Maintaining stable thermal conditions in refrigerated transport is essential for preserving temperature-sensitive products [5] such as food, pharmaceuticals, vaccines, and certain chemicals. Inadequate temperature control can result in product degradation, economic losses, and potential health risks [6]. From an energy perspective, system efficiency plays a key role in determining operational cost and environmental impact. Compressor work, pressure ratio, and exergy destruction are critical parameters that must be optimized to improve overall system performance and sustainability [7].

In recent years, environmentally friendly refrigerants such as R452A have gained attention as alternatives to high-global-warming-potential [8] (GWP) fluids, particularly in transport refrigeration applications due to their favorable thermodynamic and environmental characteristics [9]. R452A is a zeotropic refrigerant mixture and does not correspond to a single molecular species; therefore, quantum chemical calculations are performed on its individual components rather than constructing a hypothetical combined molecule. In addition to macroscopic thermodynamic analyses, advanced computational methods such as Density Functional Theory (DFT) [10], performed using the Gaussian software (Revision C.01) [11] package at the CAM-B3LYP/6-311++G(d,p) [12] level of theory within a molecular-level computational framework, together with electron density topology analysis, provide molecular-level insight into thermophysical behavior. These approaches enable a deeper understanding of the relationship between electronic structure and thermodynamic performance, contributing to the development of more efficient refrigeration systems.

## 2. Computational Details

The thermodynamic and molecular-level properties of selected components of the R452A refrigerant (R32, R125, and R1234yf) were investigated using Density Functional Theory (DFT) [10] calculations. All computations were performed using the Gaussian software [11] package, and molecular structures were visualized using GaussView (Gaussian 16 Revision C.01, Gaussian, Inc., Wallingford, CT, USA). Geometry optimizations were carried out in the gas phase at the CAM-B3LYP/6-311++G(d,p) [12] level of theory, without considering external fields or environmental effects. This approach allows the intrinsic electronic and structural properties of the molecules to be analyzed independently. This isolated-molecule approach was adopted to evaluate the intrinsic properties of each refrigerant component. It should be noted that intermolecular interactions and non-ideal mixing effects cannot be described within a single-molecule Gaussian framework; therefore, they are not included in this study. Accordingly, the present computational approach is limited to isolated-molecule analysis and does not include mixture thermodynamics or non-ideal interaction effects inherent to R452A.

Thermodynamic parameters, including thermal energy, heat capacity, entropy, and rotational constants, were calculated by considering translational, rotational, and vibrational contributions. In addition, zero-point energy (ZPE) and thermal corrections to energy, enthalpy, and Gibbs free energy were included in the analysis. Furthermore, electronic structure properties such as HOMO–LUMO energies and global reactivity descriptors were determined. Topological analysis of the electron density was performed within the framework of the Quantum Theory of Atoms in Molecules (QTAIM) to investigate bonding characteristics and charge distribution. QTAIM analyses were performed using Multiwfn version 3.7 based on the formatted checkpoint (.fchk) files generated from Gaussian calculations. Bond critical points (BCPs) were systematically located using the topology analysis module implemented in Multiwfn (developed by Tian Lu, Beijing Kein Research Center for Natural Sciences, Beijing, China). To guarantee the mathematical completeness of the critical point search, the PoincareHopf relationship was verified and fully satisfied for all investigated structures. The corresponding electron density *ρ*(r), Laplacian ∇^2^*ρ*(r), and total energy density H(r) values were evaluated at each BCP. The electron density and Laplacian contour maps presented in this work were also generated using Multiwfn. This combined approach enables the correlation of molecular-level electronic structure with macroscopic thermodynamic behavior in vapor compression refrigeration systems.

## 3. Results and Discussion

### 3.1. Analysis of Molecular Structure

R452A is a zeotropic refrigerant mixture widely used in vapor compression refrigeration systems for refrigerated transport applications. It consists of hydrofluorocarbon (HFC) and hydrofluoroolefin (HFO) components with different mass fractions, which results in non-ideal mixing behavior and composition-dependent thermophysical properties. The main components relevant to the molecular-level analysis are difluoromethane (R32), pentafluoroethane (R125), and 2,3,3,3-tetrafluoropropene (R1234yf), which exhibit distinct bonding characteristics and electronic structures.

Due to the multicomponent and non-crystalline nature of R452A, a unique crystal structure cannot be defined. Therefore, molecular-level analyses were performed using experimentally reported geometries of the individual pure components obtained from the Cambridge Structural Database (CSD/CCDC) and validated literature sources [13]. To clarify the scope of this study, it should be emphasized that the use of pure refrigerants (R32, R125, and R1234yf) does not aim to replace full mixture-property-based simulations of R452A. Instead, these components are analyzed as representative molecular constituents of the blend in order to provide fundamental insight into their individual thermodynamic, electronic, and bonding characteristics. Therefore, the objective of this work is a comparative and molecular-level interpretation rather than a direct simulation of mixture thermophysical properties.

**Figure 1:**
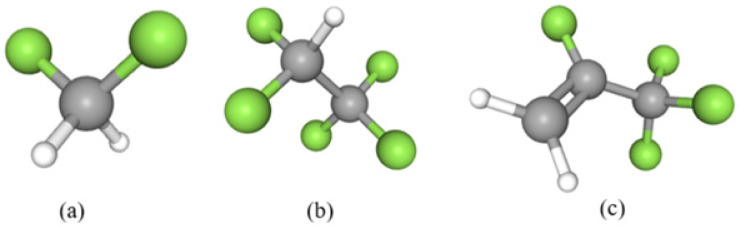
Molecular structures of the individual R452A refrigerant components: (**a**) R32, (**b**) R125, and (**c**) R1234yf, derived from X-ray crystallographic data reported in the Cambridge Structural Database (CSD; Cambridge Crystallographic Data Centre, Cambridge, UK).

The optimized geometries of R32, R125, and R1234yf were used as representative models for quantum chemical and topological analyses within the framework of Density Functional Theory (DFT). Geometry optimizations were performed to obtain stable minimum-energy structures, which were then used for electronic structure evaluation and electron density analysis. These structures provide a consistent basis for investigating bonding characteristics, molecular stability, and electron density distribution using DFT and QTAIM-based topological methods. The molecular structures used in this study are illustrated in Figure 1.

The molecular structures of the main components of the R452A refrigerant (R32, R125, and R1234yf), relevant to vapor compression refrigeration systems used in refrigerated transport, were constructed based on experimentally reported geometries and literature data, as shown in Figure 1. Each molecule was subjected to geometry optimization to obtain stable configurations corresponding to minimum-energy structures.

**Figure 2:**
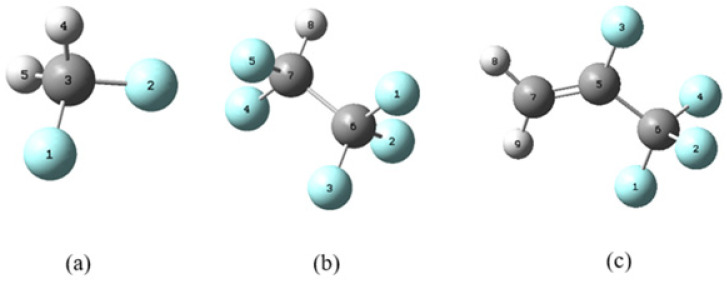
Optimized molecular structures of the individual R452A refrigerant components: (**a**) R32, (**b**) R125, and (**c**) R1234yf, calculated at the CAM-B3LYP/6-311++G(d,p) level of theory.

All molecular models were generated using the GaussView program and optimized in the gas phase at the ground state within the framework of Density Functional Theory (DFT). Calculations were performed using a hybrid exchange–correlation functional, with no external field or environmental effects included. This approach enables the intrinsic electronic and structural properties of isolated refrigerant molecules to be analyzed independently of intermolecular interactions and operating conditions. The optimized molecular geometries used in this study are presented in Figure 2.

In reference to the optimized molecular geometries presented in Figure 2, the bond lengths and bond angles obtained from DFT/CAM-B3LYP/6-311++G(d,p) calculations for the main components of the R452A refrigerant (R32, R125, and R1234yf) show good agreement with available experimental and literature-based structural data. This confirms that the optimized geometries reliably reproduce the expected molecular structural features.

Furthermore, the results indicate that the symmetry and structural regularity of each component, in terms of bond lengths and bond angles, are consistent with reported molecular structures. The calculated geometric parameters for the R452A refrigerant components are summarized in Table 1.

The comparison of experimental and optimized geometric parameters for the main components of the R452A refrigerant (R32, R125, and R1234yf), as presented in Table 1, indicates good agreement between calculated bond lengths and bond angles and available experimental or literature-based structural data. The relatively small deviations between experimental and theoretical values demonstrate that the employed DFT/CAM-B3LYP/6-311++G(d,p) level of theory provides a reliable description of the molecular geometries.

For R32, deviations in C–F bond lengths are minimal, while F–C–F bond angles show only slight differences, confirming that the optimized structure closely reproduces the expected molecular geometry. In the case of R125, calculated C–F bond lengths and bond angles also exhibit very small deviations from reported values, generally within acceptable chemical accuracy limits. Similarly, for R1234yf, both bond lengths and bond angles show good agreement with reference data, with only minor variations attributed to the presence of the unsaturated C=C bond and its associated structural flexibility.

Overall, the optimized geometries of the R452A refrigerant components are in strong agreement with experimental structural data, confirming the reliability of the chosen computational approach for describing molecular structure and bonding characteristics.

### 3.2. Investigation of Thermodynamic Properties

**Figure 3:**
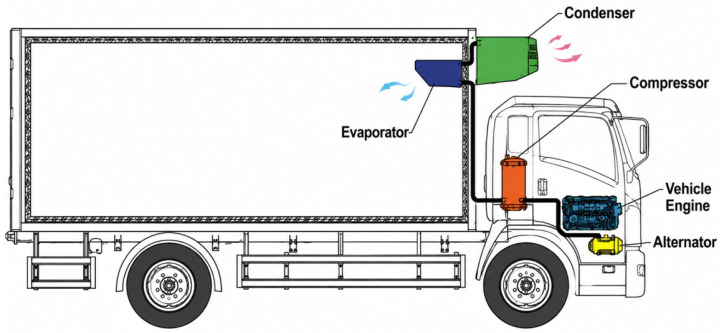
Frigorific vehicle used in cold-chain logistics for temperature-sensitive product transportation [14].

Refrigeration systems play a crucial role in cold-chain logistics, particularly in the transportation of temperature-sensitive goods. In this context, vapor compression refrigeration systems are widely used in refrigerated trucks due to their reliability and operational efficiency (Figure 3).

In this study, the thermodynamic properties of selected components of the R452A refrigerant, namely R32, R125, and R1234yf, were theoretically investigated using the Gaussian program within the framework of Density Functional Theory (DFT) at the CAM-B3LYP/6-311++G(d,p) level of theory. These components were selected as representative constituents of the R452A mixture in order to provide molecular-level insight into their thermodynamic behavior and their contribution to the overall performance of vapor compression refrigeration systems used in refrigerated trucks. The cycle performance results reported for the pure refrigerants (R32, R125, and R1234yf) are included solely for comparative molecular-level analysis and to provide insight into the influence of individual molecular characteristics on thermodynamic trends. These results do not represent realistic operational conditions of the R452A mixture, which exhibits non-ideal multicomponent behavior. Therefore, direct quantitative comparison between pure fluids and the R452A blend should not be interpreted as an actual prediction of mixture cycle performance, but rather as a qualitative framework to understand the influence of molecular structure on thermodynamic tendencies.

The calculations were performed considering fundamental thermodynamic parameters, including thermal energy, heat capacity, entropy, and rotational constants. The contributions from translational, rotational, and vibrational molecular motions were evaluated separately, and total thermodynamic functions such as internal energy, enthalpy, and entropy were subsequently determined. In addition, thermal corrections, including zero-point energy (ZPE), energy corrections, enthalpy corrections, and Gibbs free energy corrections, were taken into account. All calculated thermodynamic parameters are summarized in Table 2.

Table 2 summarizes the thermodynamic properties of the selected R452A refrigerant components (R32, R125, and R1234yf) calculated at the DFT/CAM-B3LYP/6-311++G(d,p) level of theory. For all molecules, translational and rotational energy contributions are identical, as expected for gas-phase calculations performed under identical thermodynamic conditions. The presented molecular-level results should be interpreted as intrinsic properties of individual components rather than a direct simulation of the mixture behavior.

In contrast, the vibrational energy contribution shows clear variation among the studied components, leading to differences in total thermal energy. R32 exhibits the lowest vibrational and total thermal energy values, while R1234yf shows the highest values, indicating a stronger contribution of vibrational modes due to increased molecular size and structural flexibility. R125 lies between these two extremes, reflecting intermediate molecular complexity.

A similar trend is observed in heat capacity and entropy values. Both properties increase with molecular size and structural complexity, where vibrational contributions dominate the total thermodynamic quantities. In particular, R1234yf presents the highest entropy, while R32 exhibits the lowest, consistent with its more compact and symmetric molecular structure.

Rotational constants and rotational temperatures further highlight structural differences among the components. Smaller and more symmetric molecules such as R32 display higher rotational constants, whereas larger molecules such as R1234yf show lower values, reflecting increased moments of inertia. Overall, these results demonstrate that molecular complexity directly governs vibrational activity, entropy, and thermal properties, which play a crucial role in irreversibility generation and thermodynamic performance in vapor compression refrigeration systems.

The variation in exergy efficiency with compression ratio shows a decreasing trend for all refrigerants, indicating increasing irreversibilities at higher pressure ratios. R32 exhibits the highest exergy efficiency across the entire operating range, followed by R1234yf and R452A, while R125 shows the lowest performance. The difference between refrigerants is more pronounced at low compression ratios and gradually decreases as the compression ratio increases.

**Figure 4:**
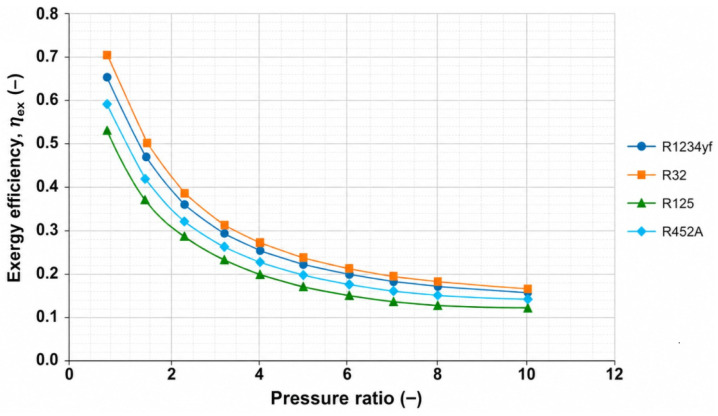
Effect of compression ratio on exergy efficiency.

Figure 4 shows that, for all fluids, exergy efficiency (ηex) decreases as the compression ratio (r) increases, indicating rising irreversibilities. When comparing the fluids, R32 exhibits the highest exergy efficiency and stands out as the most efficient option, followed by R1234yf and R452A. R125, on the other hand, shows the lowest efficiency across all r values and represents the fluid with the highest exergy loss. Furthermore, the difference between the fluids is more pronounced at low compression ratios, while the curves converge at higher r values.

**Figure 5:**
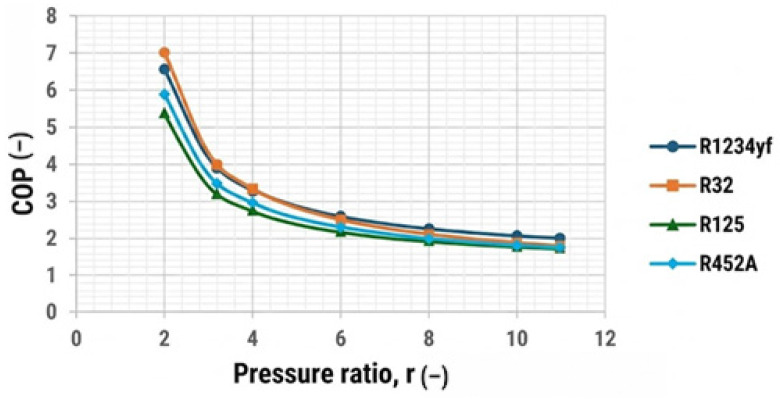
The effect of changes in the rate of pressure increase on the COP value.

Figure 5 shows that the COP decreases with increasing compression ratio (r) for all refrigerants, indicating a reduction in system performance due to higher pressure ratios. Among the working fluids, R32 exhibits the highest COP values across the entire range, followed by R1234yf and R452A, while R125 consistently shows the lowest performance. The performance gap between refrigerants is more pronounced at low compression ratios and gradually diminishes as r increases, leading to convergence of the curves at higher operating conditions.

**Figure 6:**
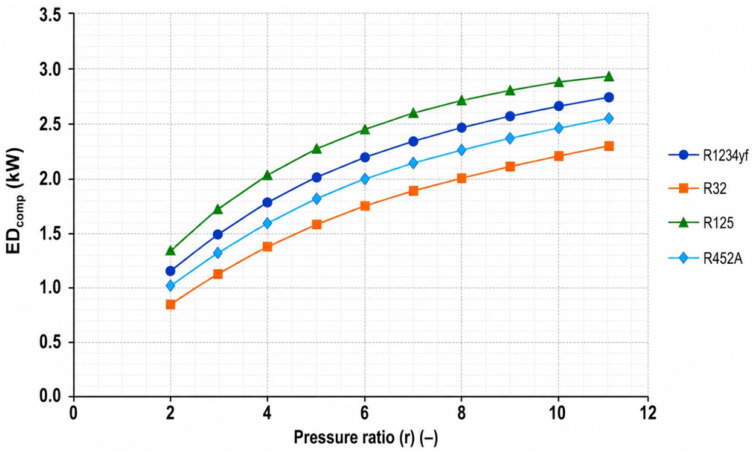
The effect of changes in the rate of pressure increase on the exergy destruction of the compressor.

As shown in Figure 6, the relationship between compressor exergy destruction and compression ratio shows that, for all fluids, an increase in compression ratio triggers irreversibilities in the system, thereby increasing exergy destruction. Among the fluids presented in the graph, R32 exhibits the most efficient performance across all operating ranges and shows low exergy destruction values. R32 is followed by R1234yf and R452A with higher loss levels, while R125 clearly results in the highest usable energy loss within the compressor. The performance difference between the fluids is particularly pronounced at lower compression ratios, highlighting the importance of selecting low-loss refrigerants such as R32 during the system design phase for improved energy efficiency.

**Figure 7:**
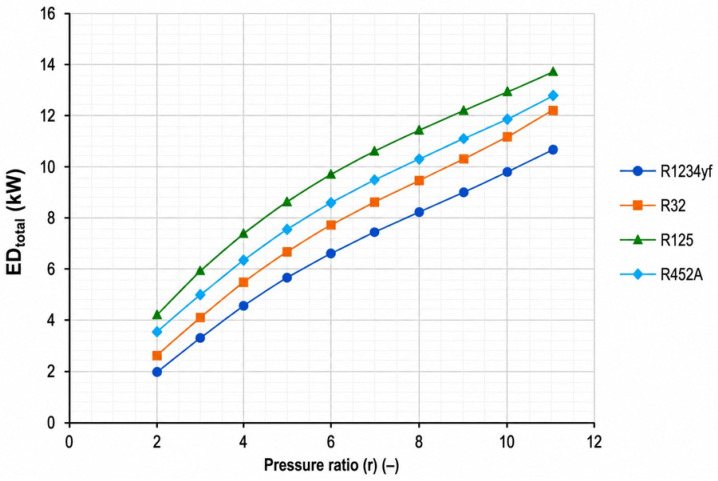
The effect of changes in the rate of pressure increase on the total exergy destruction.

Figure 7 indicates that R125 exhibits the highest exergy loss and is therefore the least efficient fluid, while R1234yf shows the most thermodynamically efficient performance with the lowest exergy destruction. R452A and R32 are positioned in the intermediate range, and the differences between the fluids become more pronounced as the pressure rise ratio increases.

**Figure 8:**
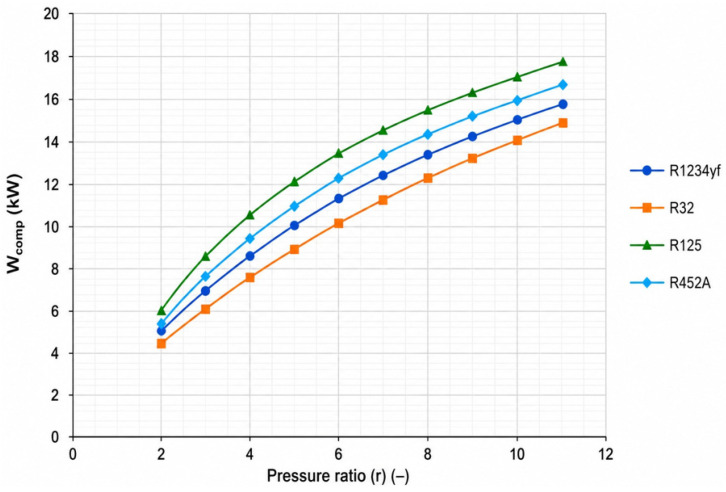
The effect of changes in the rate of pressure increase on compressor work.

Figure 8 shows that compressor work increases with increasing pressure ratio for all refrigerants. Among the studied fluids, R125 requires the highest compressor work and therefore the highest energy input. R1234yf exhibits the lowest compressor work, indicating the most favorable performance, while R452A and R32 show intermediate behavior. The difference in energy consumption among the fluids becomes more pronounced as the pressure ratio increases.

### 3.3. QTAIM Analysis of Intermolecular Interactions

Let $\rho :IR^{3}\to IR$ denote the electron density obtained from KohnSham density functional theory (DFT) calculations [15]. We assume that ρ is at least $C^{2}$ smooth and that its critical points are non-degenerate (Morse function assumption) [16], in accordance with the Atoms in Molecules (AIM) framework.

Isodensity surfaces are defined as level sets:

$$\sum_{e}=\left\{\left(x,y,z\right)\in IR^{3}\left|\rho \left(x,y,z\right)=c\right.\right\},$$

Whenever ∇ρ≠0, these sets define smooth embedded surfaces. Thus, bonding analysis becomes the study of critical points of ρ, curvature of level surfaces, and geometry of gradient flow lines.

**Figure 9:**
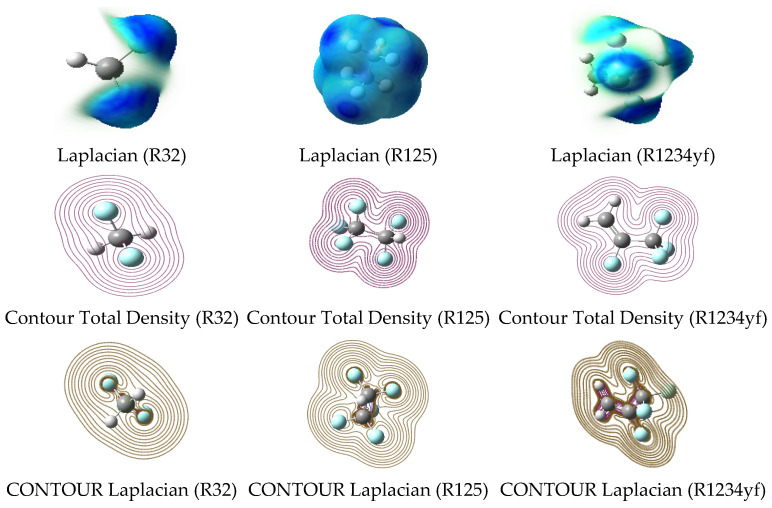
Contour maps of electron density (*ρ*) and its Laplacian (∇^2^*ρ*) for R452A refrigerant components at the DFT/CAM-B3LYP/6-311++G(d,p) level of theory.

To visualize and compare the electronic structure characteristics of the studied refrigerants, contour maps of the electron density (*ρ*) and its Laplacian (∇^2^*ρ*) were generated for R32, R125, and R1234yf within the framework of DFT calculations. The electron density contour plots provide a direct representation of charge distribution within the molecular framework, while the Laplacian of electron density highlights regions of charge concentration (∇^2^*ρ* < 0) and charge depletion (∇^2^*ρ* > 0), which are indicative of bonding and non-bonding interaction domains, respectively. These graphical representations allow a qualitative comparison of the electronic environments of the three refrigerants. The corresponding contour maps are presented in Figure 9.

The contour maps illustrate the spatial distribution of electron density (*ρ*) and its Laplacian (∇^2^*ρ*) for R32, R125, and R1234yf refrigerants. The electron density plots reflect the charge localization within the molecular framework, while the Laplacian maps identify regions of charge concentration and depletion associated with bonding and non-bonding interactions.

In addition to the qualitative visualization, a quantitative topological description of bonding has been carried out within the QTAIM framework. For this purpose, electron density (*ρ*), Laplacian (∇^2^*ρ*), and total energy density H(r) values were evaluated at bond critical points (BCPs, (3,−1)) for representative bonds.

As presented in Table 3, some C–F bonds within highly fluorinated components (specifically the CF3 groups in R125 and R1234yf) exhibit relatively large negative Laplacian ∇^2^*ρ* and total energy density H(r) values. According to the established QTAIM classification criteria by Espinosa et al. and Macchi et al., the simultaneous occurrence of ∇^2^*ρ* < 0 and H(r) < 0 denotes an intermediate (transitional) bonding region rather than a typical shared-shell covalent or purely closed-shell ionic interaction. This topological behavior is a direct consequence of the perfluorination accumulation effect, where multiple highly electronegative fluorine atoms bonded to adjacent carbon centers induce a strong charge-shift mechanism. This mechanism heavily localizes the electron density along the bond paths at the triple-zeta split-valence level with diffuse and polarization functions (CAM-B3LYP/6-311++G(d,p)). Consequently, these interactions cannot be categorized as isolated polar bonds; instead, they represent a complex intermediate structure balancing local electronic concentration with substantial ionic contributions.

The QTAIM results provide a quantitative description of bonding interactions in the studied refrigerants. While several bonds exhibit negative Laplacian values (∇^2^*ρ* < 0), indicating regions of electron concentration typical of shared-shell interactions, highly polarized bonds such as C–F display a broader range of Laplacian values. This behavior reflects the mixed character of these bonds, where significant electron sharing coexists with strong bond polarization due to the high electronegativity of fluorine atoms.

In particular, the relatively large magnitude of negative ∇^2^*ρ* values observed for some bonds may be attributed to the chosen level of theory (CAM-B3LYP) and basis set, which can enhance electron localization effects in fluorinated systems. Similar trends have been reported in theoretical studies of fluorinated hydrocarbons, where deviations from classical closed-shell behavior are observed.

Among the investigated systems, the C=C bond in R1234yf shows the highest electron density and most negative energy density, indicating strong electron sharing. In contrast, C–F bonds, particularly in R125, exhibit more polarized electron density distributions, reflecting partial electrostatic character due to fluorine substitution. These differences in electron density topology are directly related to molecular polarization and are expected to influence intermolecular interactions and thermodynamic behavior.

These QTAIM findings provide insight into the molecular-level origins of macroscopic thermodynamic behavior. In particular, the degree of electron localization and bond polarization, especially in C–F bonds, directly influences molecular polarity and intermolecular interactions. Refrigerants with higher bond polarization and electron density localization tend to exhibit stronger intermolecular forces, which can affect key thermodynamic properties such as enthalpy of vaporization and heat transfer characteristics. Consequently, the observed differences in QTAIM parameters among R32, R125, and R1234yf may contribute to variations in cycle performance metrics such as COP and exergy efficiency.

Bonding analysis is based on the topology of the electron density within the QTAIM framework and is further complemented by orbital-based electronic structure analysis in the following subsection.

For example, the stronger electron density concentration and more negative H(r) values observed for C–F bonds in R125 are consistent with its higher molecular stability and lower flammability compared with less fluorinated refrigerants. Previous studies have shown that increased bond polarization and electron localization in fluorinated hydrocarbons are associated with reduced chemical reactivity and altered thermodynamic behavior, including changes in enthalpy and intermolecular interactions. In this context, the QTAIM descriptors obtained in the present study provide a molecular-level interpretation of the thermodynamic characteristics of the investigated refrigerants.

The calculated electronic structure parameters presented in Table 4 provide detailed insight into the relative stability and chemical reactivity of the selected R452A refrigerant components. The HOMO energy values indicate that R32 exhibits the lowest tendency for electron donation, while R1234yf shows a higher propensity for electron transfer processes. Similarly, the LUMO energies suggest that R1234yf has a greater ability to accept electrons compared to R32 and R125, indicating enhanced reactivity.

The HOMO–LUMO [17] energy gap (ΔE) distinguishes the electronic stability of the investigated molecules. R32 exhibits the largest energy gap (10.84 eV), indicating higher chemical stability, whereas R1234yf shows the smallest gap (6.15 eV), reflecting increased chemical reactivity. R125 lies between these two extremes, demonstrating intermediate electronic stability.

Global reactivity descriptors [18] further support these trends. Chemical hardness (η) decreases from R32 to R1234yf, while chemical softness (S) increases accordingly, indicating reduced molecular rigidity with increasing fluorinated olefin character. The electrophilicity index (ω) is higher for R1234yf, suggesting a stronger tendency to undergo electrophilic interactions compared to the other components.

Overall, these results demonstrate a correlation between molecular electronic structure and reactivity, where saturated molecules such as R32 exhibit higher stability, while unsaturated HFO-type molecules such as R1234yf display enhanced chemical reactivity. These differences are expected to influence the thermodynamic behavior and performance characteristics of the R452A refrigerant system.

**Figure 10:**
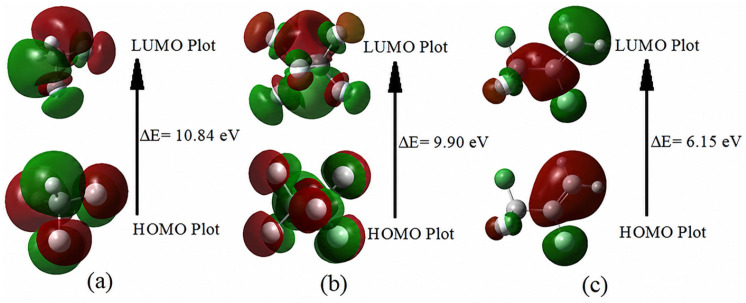
HOMO and LUMO isosurfaces of the individual R452A refrigerant components: (**a**) R32, (**b**) R125, and (**c**) R1234yf, calculated at the DFT/CAM-B3LYP/6-311++G(d,p) level of theory.

For the visualization of HOMO–LUMO distributions, two representative components exhibiting extreme electronic behavior within the R452A system were selected. Accordingly, R32 (widest energy gap and highest stability) and R1234yf (narrower gap and higher reactivity) were analyzed, and their HOMO and LUMO isosurfaces are presented in Figure 10.

The HOMO and LUMO isosurfaces presented in Figure 10 reveal distinct electronic characteristics for R32 and R1234yf. The positive and negative phases of the molecular orbitals are represented by different colors, illustrating the spatial distribution of electron density.

For R32, the HOMO is mainly localized around the C–F bonding regions [19], indicating a more localized electron distribution and higher electronic stability. In contrast, for R1234yf, both HOMO and LUMO orbitals are more delocalized over the molecular framework, particularly around the unsaturated C=C bond, which reflects increased electron delocalization and higher chemical reactivity.

The HOMO–LUMO energy gap obtained from these isosurfaces provides a direct measure of molecular stability, where larger gaps indicate higher kinetic stability and lower chemical reactivity, whereas smaller gaps correspond to increased reactivity. This behavior is consistent with global reactivity descriptors, including chemical hardness and electrophilicity index, as well as with recent literature on the relationship between frontier orbital energies and molecular stability, reflecting the influence of electronic structure on the thermodynamic and operational behavior of refrigerant molecules.

#### 3.3.1. Local Structure at Bond Critical Points

A bond critical point (BCP) satisfies

$$\nabla \rho \left(\tau_{BCP}\right)=0.$$

Let $H_{\rho }$ denote the Hessian matrix of ρ evaluated at the BCP. Under QTAIM conditions, its eigenvalue signature is

$$\left(\lambda_{1},\lambda_{2},\lambda_{3}\right)=\left(-,-,+\right),$$

(see, [20]). By the Morse lemma, the local behavior of the electron density near the BCP can be expressed as

$$\rho =\rho_{0}+\frac{1}{2}\left(\lambda_{1}u_{1}^{2}+\lambda_{2}u_{2}^{2}+\lambda_{3}u_{3}^{2}\right),$$

where $\rho_{0}=\rho \left(\tau_{BCP}\right)$ and $\left(u_{1},u_{2},u_{3}\right)$ are coordinates aligned with the Hessian eigenvectors.

The directions $u_{1}$ and $u_{2}$ correspond to local charge concentration, while $u_{3}$ corresponds to local charge depletion. The bond path tangent aligns with the eigenvector associated with the positive eigenvalue $\lambda_{3}$.

#### 3.3.2. Curvature of Isodensity Surfaces

For regular values of ρ, the mean curvature of the level surface satisfies

$$H=\frac{1}{2}\nabla \left(\frac{\nabla \rho }{\left|\nabla \rho \right|}\right).$$

Near a BCP, curvature anisotropy is governed by the Hessian eigenvalues. We define a transverse curvature anisotropy parameter:

$$\eta =\frac{\left|\lambda_{1}\right|-\left|\lambda_{2}\right|}{\left|\lambda_{1}\right|+\left|\lambda_{2}\right|}.$$

When η=0, transverse charge accumulation is isotropic; when η→1, bonding becomes highly directional. This quantity serves as a quantitative geometric descriptor of bond character.

#### 3.3.3. Differential Geometry of Bond Path

Bond paths are integral curves of the gradient vector field:

$$\frac{d\gamma }{ds}=\nabla \rho \left(\gamma (s)\right),$$

where s denotes arc length [20]. These curves are generally not straight lines. Introducing the Frenet–Serret frame $\left\{T,N,B\right\}$ along the bond path, the curvature is given by

$$\kappa =\left|\frac{dT}{ds}\right|,$$

(see, [21]). A small curvature κ indicates a rigid and strongly directed bond, whereas larger curvature reflects weaker directional confinement. At the BCP,

$$T//e_{3}$$

where $e_{3}$ is the eigenvector corresponding to the positive eigenvalue $\lambda_{3}$ of the Hessian. Thus, the local topology of the electron density and the global geometry of the bond path are intrinsically linked.

#### 3.3.4. Comparative Geometric Analysis

##### R32 (Difluoromethane)

R32 exhibits polar C--F bonds. Large magnitudes of $\lambda_{1}$ and $\lambda_{2}$ are expected, together with a positive Laplacian at the BCP. The corresponding isodensity shells tend to be convex, reflecting closed-shell characteristics and strong transverse curvature concentration.

##### R125 (Pentafluoroethane)

In R125, increased fluorination leads to stronger localization of electron density. Larger Laplacian magnitudes and increased curvature anisotropy are anticipated, indicating enhanced charge confinement around nuclei.

##### R1234yf (2,3,3,3-Tetrafluoropropene)

R1234yf contains a C=C double bond with π-electron delocalization. A negative Laplacian at the BCP of the double bond is expected, reflecting shared-shell character. Geometrically, this corresponds to reduced transverse curvature and a more symmetric saddle structure.

#### 3.3.5. ThermodynamicGeometric Correspondence

Bond stiffness relates to second derivatives of the total energy:

$$k\sim \frac{\partial^{2}E}{\partial r^{2}}.$$

Since local density curvature reflects electronic confinement, we propose the correspondence

$$k\sim \lambda_{3},$$

where $\lambda_{3}$ is the positive Hessian eigenvalue at the BCP.

Consequently, larger $\lambda_{3}$ values imply higher vibrational frequencies,

$$\nu \sim \sqrt{k}$$

which influence entropy and heat capacity contributions. This establishes a geometric bridge between curvature descriptors of the electron density and thermodynamic stability.

#### 3.3.6. Example (Local Differential Geometry at a Model BCP):

We consider a model electron density function mimicking the characteristics of a bond critical point (BCP):

$$\rho \left(x,y,z\right)=\rho_{0}-x^{2}-(0,6)y^{2}+(0,8)z^{2},$$

where $\rho_{0}$ is the density at the BCP located at the origin. This function has the Hessian eigenvalue signature $\left(-,-,+\right)$, corresponding to a saddle point: two directions of local charge accumulation (x and y axes) and one direction of depletion (z axis, aligned with the bond path).

**Figure 11:**
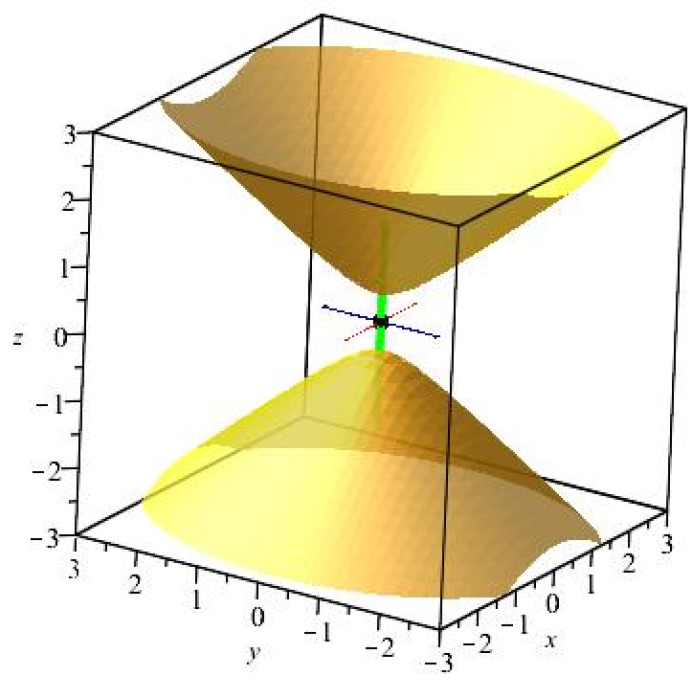
Isosurface of the model electron density at $\rho =\rho_{0}+\varepsilon$ with mean curvature mapped in color. Red and blue arrows indicate the transverse Hessian eigenvectors; green arrow indicates the bond-path direction (positive eigenvalue).

Figure 11 illustrates the saddle-point topology of a QTAIM bond critical point. The mean curvature distribution highlights regions of local convexity and concavity, while the eigenvectors indicate the principal directions of charge concentration (transverse) and depletion (bond path direction). This representation provides a geometric interpretation of bonding consistent with QTAIM theory.

#### 3.3.7. Quantitative Geometric Descriptors

To establish a concrete comparison between the fluorinated refrigerants, we introduce quantitative geometric descriptors derived from the Hessian eigenvalues at the bond critical points.

Let $\left(\lambda_{1},\lambda_{2},\lambda_{3}\right)$ denote the ordered eigenvalues of the Hessian matrix of the electron density at a BCP, with $\lambda_{1}\leq \lambda_{2}<0<\lambda_{3}$.

The total transverse curvature concentration is defined as

$$C_{⊥}=\left|\lambda_{1}\right|+\left|\lambda_{2}\right|.$$

This quantity measures the degree of electron density accumulation in the plane perpendicular to the bond path.

To quantify directional asymmetry in transverse confinement, we define

$$\eta =\frac{\left|\lambda_{1}\right|-\left|\lambda_{2}\right|}{\left|\lambda_{1}\right|+\left|\lambda_{2}\right|}.$$

Here, 0≤η<1.

Small values of η indicate isotropic accumulation, whereas larger values reflect directional polarization effects.

The longitudinal depletion strength is characterized by the positive eigenvalue

$$C_{\|}=\lambda_{3}.$$

This quantity governs the resistance to electron accumulation along the bond path and is directly related to bond stiffness.

We propose a dimensionless geometric rigidity index

$$R=\frac{\lambda_{3}}{\left|\lambda_{1}\right|+\left|\lambda_{2}\right|}.$$

Larger values of R correspond to stronger shared-shell interactions and enhanced bond rigidity, whereas smaller values reflect closed-shell character.

These descriptors provide a unified geometric framework for systematic comparison of bonding properties across R32, R125, and R1234yf.

## 4. Conclusions

In this study, a multiscale approach was employed to investigate the R452A refrigerant used in refrigerated transport systems. Thermodynamic and exergy analyses were performed to evaluate the performance of the vapor compression refrigeration cycle and to identify irreversibilities within the system.

At the molecular level, Density Functional Theory (DFT) calculations were used to examine the electronic structure and stability of the refrigerant components. In addition, a differential geometric framework based on electron density topology was applied to analyze bonding characteristics in terms of curvature and bond critical point properties.

The results show that local geometric descriptors, such as Hessian eigenvalues and curvature-based indices, are strongly correlated with bond strength and molecular stability. The QTAIM-based analysis also provides a clear visualization of curvature anisotropy and bonding directionality, offering a physically consistent interpretation of electronic structure.

Overall, the integration of thermodynamic analysis with molecular-scale geometric descriptors establishes a clear relationship between microscopic electronic structure and macroscopic refrigeration performance. This combined framework provides a useful tool for evaluating and comparing refrigerants in energy and environmental applications.

The findings represent component-level molecular insights rather than a full thermodynamic representation of the R452A mixture.

## Figures and Tables

**Table 1:** Experimental and optimized geometric parameters of the R452A refrigerant components calculated at the DFT/CAM-B3LYP/6-311++G(d,p) level of theory.

R32			R125			R1234yf		
Bond Length (Å)	Exp.	Opt.	Bond Length (Å)	Exp.	Opt.	Bond Length (Å)	Exp.	Opt.
F1-C3	1.35394	1.35886	F1-C6	1.34971	1.33541	F1-C6	1.32515	1.33716
F2-C3	1.35394	1.35886	F3-C6	1.34913	1.32835	F2-C6	1.34984	1.34017
			F2-C6	1.34969	1.33544	F4-C6	1.33913	1.33967
			C6-C7	1.50023	1.53300	C6-C5	1.48978	1.50318
			F4-C7	1.35532	1.34780	C5-C7	1.30474	1.31325
			F5-C7	1.35526	1.34779	F3-C5	1.34329	1.33863
Bond Angles (^o^)	Exp.	Opt.	Bond Angles (^o^)	Exp.	Opt.	Bond Angles (^o^)	Exp.	Opt.
F1-C3-F2	107.08964	108.36881	F1-C6-F2	107.77946	108.38561	F1-C6-F2	107.63666	107.96071
			F1-C6-F3	108.02323	108.80977	F1-C6-F4	107.90487	107.99099
			F2-C6-F3	108.01550	108.80913	F1-C6-C5	112.04216	111.12687
			F1-C6-C7	110.88049	109.58249	F2-C6-F4	105.91733	107.28737
			F2-C6-C7	110.87832	109.57722	F2-C6-C5	111.31625	111.14610
			F4-C7-F5	106.93466	108.78087	C6-C5-F3	109.93571	110.95739
						C6-C5-C7	126.46563	126.08858

**Table 2:** Thermodynamic parameters of the R452A refrigerant components calculated at the DFT/CAM-B3LYP/6-311++G(d,p) level of theory.

	R32	R125	R1234yf
Thermal energy, E (Kcal/mol)			
Rotational	0.889	0.889	0.889
Translational	0.889	0.889	0.889
Vibrational	20.839	25.817	33.402
Total	22.616	27.595	35.180
Heat capacity, C_v_ (cal/mol K)			
Rotational	2.981	2.981	2.981
Translational	2.981	2.981	2.981
Vibrational	2.255	14.393	15.750
Total	8.217	20.355	21.712
Entropy, S (cal/mol K)			
Rotational	20.350	27.292	27.259
Translational	37.769	40.261	40.109
Vibrational	0.805	12.140	12.618
Total	58.925	79.694	79.986
Rotational constants (GHz)			
A	49.35707	3.68473	3.71561
B	10.56928	2.41478	2.47110
C	9.24006	2.00141	2.00515
Rotational temperature (K)			
A	2.36877	0.17684	0.17832
B	0.50725	0.11589	0.11859
C	0.44345	0.09605	0.09623
Thermal properties (Hartree/particle)			
Zero-point correction	0.032917	0.037872	0.049802
Thermal correction to Energy	0.036041	0.043975	0.056063
Thermal correction to Enthalpy	0.036985	0.044919	0.057007
Thermal correction to Gibbs Free Energy	0.008988	0.007054	0.019003
Sum of electronic and zero-point Energies	−238.965839	−576.030167	−514.833425
Sum of electronic and thermal Energies	−238.962715	−576.024065	−514.827165
Sum of electronic and thermal Enthalpies	−238.961771	−576.023120	−514.826221
Sum of electronic and thermal Free Energies	−238.989768	−576.060986	−514.864225
Zero point vibrational energy (kcal/mol)	20.65602	23.76503	31.25144

**Table 3:** Quantitative QTAIM topological parameters calculated at selected bond critical points (BCPs) for R452A refrigerant components at the DFT/CAM-B3LYP/6-311++G(d,p) level of theory.

Molecule	Bond (BCP, (3,−1))	CP Index	*ρ* (a.u.)	∇^2^*ρ* (a.u.)	H(r) (a.u.)	Bond Character
R32 (CH_2_F_2_)	C–H	CP 7/CP 8	0.2796	−0.8682	−0.2495	Shared-shell (Covalent)
	C–F	CP 6/CP 9	0.2353	−0.1766	−0.2536	Intermediate (Polar covalent)
R125 (C_2_HF_5_)	C–H	CP12	0.2860	−0.2199	−0.4039	Shared-shell (Covalent)
	C–F(CF3 group)	CP9/CP 14	0.2707	−0.7228	−0.2296	Intermediate (Charge-shift)
	C–C	CP10/CP 15	0.2960	−1.0854	−0.2976	Shared-shell (Covalent)
	C–F(CF2 group)	CP11/CP 13	0.2657	−0.0924	−0.3625	Intermediate (Strong ionic contribution)
R1234yf	C–H	CP11	0.2402	−0.2107	−0.2697	Shared-shell (Covalent)
	C–F(CF3 group)	CP10/CP 12	0.2506	−0.7147	−0.2319	Intermediate (Charge-shift)
	C=C	CP14	0.2763	−0.8379	−0.2319	Shared-shell (Double bond character)
	C–F(on C=C)	CP13	0.2415	−0.1997	−0.2713	Intermediate (Polar covalent)

CP index denotes the labeling of bond critical points identified in the QTAIM analysis.

**Table 4:** Theoretically calculated electronic structure parameters of the R452A refrigerant components at the DFT/CAM-B3LYP/6-311++G(d,p) level of theory.

Parameters	R32	R125	R1234yf
E_HOMO_ (eV)	−9.78	−9.12	−9.44
E_LUMO_ (eV)	1.06	0.78	−3.29
ΔE = E_LUMO_ − E_HOMO_ (eV)	10.84	9.90	6.15
I (eV)	9.78	9.12	9.44
A (eV)	−1.06	−0.78	3.29
μ (eV)	−4.36	−4.17	−6.37
χ (eV)	4.36	4.17	6.37
η (eV)	5.42	4.95	3.08
S (eV^−1^)	0.092	0.101	0.163
ω (eV)	1.75	1.76	6.60
E_TOTAL_ (Hartree)	−238.99876	−576.06804	−514.88323

## Data Availability

All data generated or analyzed during this study are included in this published article. Additional data are available from the corresponding author upon reasonable request.

## Abbreviations

R452A	Low-flammability zeotropic refrigerant blend consisting of R32, R125, and R1234yf
R32	Difluoromethane
R125	Pentafluoroethane
R1234yf	2,3,3,3-Tetrafluoropropene
COP	Coefficient of Performance (Performans Katsayısı)
DFT	Density Functional Theory (Yoğunluk Fonksiyoneli Teorisi)
CAM-B3LYP	Coulomb-attenuating method hybrid exchange–correlation functional
6-311++G(d,p)	Triple-zeta basis set with diffuse and polarization functions
QTAIM/AIM	Quantum Theory of Atoms in Molecules (Atoms in Molecules Theory)
ELF	Electron Localization Function (Elektron Lokalizasyon Fonksiyonu)
RDG	Reduced Density Gradient (İndirgenmiş Yoğunluk Gradyanı)
HOMO	Highest Occupied Molecular Orbital (En Yüksek Dolu Moleküler Orbital)
LUMO	Lowest Unoccupied Molecular Orbital (En Düşük Boş Moleküler Orbital)
ΔE	HOMO–LUMO energy gap (enerji aralığı)
η	Chemical hardness (kimyasal sertlik)
S	Chemical softness (kimyasal yumuşaklık)
ω	Electrophilicity index (elektrofiliklik indeksi)
μ	Chemical potential (kimyasal potansiyel)
χ	Electronegativity (elektronegatiflik)
ED	Exergy Destruction (Ekserji yıkımı)
η (exergy efficiency)	Exergy efficiency (ekserji verimi)
W	Compressor work (Kompresör işi)
$\dot{Q}$cond	Heat rejected in condenser (kondenser ısı atımı)
$\dot{Q}$cool	Cooling capacity (soğutma kapasitesi)
GWP	Global Warming Potential (Küresel Isınma Potansiyeli)
HFC	Hydrofluorocarbon (Hidroflorokarbon)
HFO	Hydrofluoroolefin (Hidrofloroolefin)
CFC	Chlorofluorocarbon (Kloroflorokarbon)
HCFC	Hydrochlorofluorocarbon (Hidrokloroflorokarbon)
ZPE	Zero-Point Energy (Sıfır Nokta Enerjisi)
BCP	Bond Critical Point (Bağ Kritik Noktası)
H(r)	Total energy density (toplam enerji yoğunluğu)

## References

[1] Çengel Y.A., Boles M.A. (2015). Thermodynamics: An Engineering Approach.

[2] Dincer I., Rosen M.A. (2013). Exergy: Energy, Environment and Sustainable Development.

[3] Moran M.J., Shapiro H.N., Boettner D.D., Bailey M.B. (2014). Fundamentals of Engineering Thermodynamics.

[4] Bejan A. (2016). Advanced Engineering Thermodynamics.

[5] James S.J., James C. (2010). The food cold-chain and climate change. Food Res. Int..

[6] Aung M.M., Chang Y.S. (2014). Temperature management for the quality assurance of a perishable food supply chain. Food Control.

[7] Sarkar J. (2015). Performance of transcritical CO_2_ refrigeration cycles: A review. Renew. Sustain. Energy Rev..

[8] Mota-Babiloni A., Navarro-Esbrí J., Makhnatch P., Molés F. (2017). R32 as a low GWP working fluid in refrigeration systems: A review. Int. J. Refrig..

[9] Minor B., Spatz M. (2014). HFO refrigerants: Low GWP alternatives. Int. J. Refrig..

[10] Kohn W., Sham L.J. (1965). Self-consistent equations including exchange and correlation effects. Phys. Rev..

[11] Frisch M.J., Trucks G.W., Schlegel H.B., Scuseria G.E., Robb M.A., Cheeseman S.R., Scalmani G., Barone V., Petersson G.A., Nakatsuji H. (2016). Gaussian 16 Revision C.01.

[12] Yanai T., Tew D.P., Handy N.C. (2004). A new hybrid exchange–correlation functional using the Coulomb-attenuating method (CAM-B3LYP). Chem. Phys. Lett..

[13] Groom C.R., Bruno I.J., Lightfoot M.P., Ward S.C. (2016). The Cambridge Structural Database. Acta Crystallogr. Sect. B.

[14] Keven A., Cimşit C., Bardak S., Ayata Ü. (2023). The analysis of the refrigeration system of frigorific vehicles used in road transport. Current Research in Engineering.

[15] Zhang Z., Liu L., Glenn D.M., Jana A., Mora Perez C., Qian J. (2025). Real-space Kohn–Sham density functional theory for complex energy applications. Chem. Commun..

[16] Milnor J. (1963). Morse Theory.

[17] Gümüş H.P., Tamer Ö., Avcı D., Atalay Y. (2015). A theoretical study on 2-chloro-5-(2-hydroxyethyl)-4-methoxy-6-methylpyrimidine by DFT/ab initio calculations. Mater. Sci..

[18] Gümüş H. (2020). Conformational, spectroscopic, electric and electronic investigations on 5-nitropyridine-2-hydrazino-3-carbonitrile-6-methyl-4-(methoxymethyl): Molecular docking study. J. Mol. Struct..

[19] Gümüş H. (2020). Spectroscopic (Vibrational and NMR) characterizations and molecular docking analysis of Zn(II), Cd(II) and Hg(II) complexes with alkyl-aryl dithiocarbamates. Arab. J. Sci. Eng..

[20] Bader R.F.W. (1990). Atoms in Molecules: A Quantum Theory.

[21] do Carmo M.P. (1976). Differential Geometry of Curves and Surfaces.

